# *In vitro* antibacterial activity of different pulp capping materials

**DOI:** 10.4317/jced.52401

**Published:** 2015-12-01

**Authors:** Claudio Poggio, Riccardo Beltrami, Marco Colombo, Matteo Ceci, Alberto Dagna, Marco Chiesa

**Affiliations:** 1 Department of Clinical-Surgical, Diagnostic and Pediatric Sciences – Section of Dentistry, University of Pavia, Piazzale Golgi 3, 27100 Pavia, Italy; 2 Department of Brain and Behavioural Sciences - Section of Statistic, University of Pavia, Piazzale Golgi 3, 27100 Pavia, Italy

## Abstract

**Background:**

Direct pulp capping involves the application of a dental material to seal communications between the exposed pulp and the oral cavity (mechanical and carious pulp exposures) in an attempt to act as a barrier, protect the dental pulp complex and preserve its vitality. The aim of this study was to evaluate and compare, by the agar disc diffusion test, the antimicrobial activity of six different pulp-capping materials: Dycal (Dentsply), Calcicur (Voco), Calcimol LC (Voco), TheraCal LC (Bisco), MTA Angelus (Angelus), Biodentine (Septodont).

**Material and Methods:**

*Streptococcus salivarius*, *Streptococcus sanguis* and *Streptococcus mutans* strains were selected to evaluate the antimicrobial activity by the agar disc diffusion test of different pulp capping materials. Paper disks were impregnated whit each pulp capping materials and placed onto culture agar-plates pre-adsorbed with bacterial cells and further incubated for 24 h at 37°C. The growth inhibition zones around each pulp capping materials were recorded and compared for each bacterial strain.

**Results:**

For the investigation of the antibacterial properties the ANOVA showed the presence of significant differences among the various materials. Tukey test showed that MTA-based materials induced lower growth inhibition zones.

**Conclusions:**

MTA-based products show a discrete antibacterial activity varying from calcium hydroxide-based materials which present an higher antibacterial activity.

** Key words:**Agar disc diffusion test, antimicrobial activity, calcium hydroxide, MTA, pulp capping materials.

## Introduction

Pulp capping procedure, consisting in cover the exposed pulp with a suitable dental material in order to protect the dental pulp complex and preserve its vitality, is frequently performed in dental practice. On cariously and mechanically exposed teeth, this treatment can be considered temporary. Therefore, according to various Authors (1,2) the vital-pulp therapy treatment could be permanent.

Stanley (1) stated that, in case of accidentally carious exposure on an asymptomatic pulp, the pulp-capping procedure could be performed successfully. Haskell *et al.* (2) estimated a 12-year survival after asymptomatic carious exposures and pulp-capping. The presence of microorganisms played a fundamental role in the development and progression of pulpal and periapical disease and pulp-capping failures (3). A bactericidal material could make pulp-capping treatments long-term.

Several materials such as calcium hydroxide-based materials and more recently mineral trioxide aggregate (MTA) are commonly used as pulp-capping agents. Calcium hydroxide is the most popular agent for direct and indirect pulp capping (4,5). The high pH (12.5) gives calcium hydroxide an important antimicrobial activity. According to Siqueira (6), the bactericidal action of Ca(OH)2 depends on the release of hydroxyl ions in an aqueous environment. Siqueira and Lopes (7) evaluated that hydroxyl ions are highly oxidant free radicals that show extreme reactivity with several biomolecules, causing denaturation of proteins and damages to the bacterial cytoplasmic membrane.

Contrariwise, conflicting reports emerge from the evaluation of the antibacterial and antifungal properties of MTA (8-10). However MTA seems to have limited antimicrobial effect against some microorganisms (11,12). Using the agar disc diffusion test, the antimicrobial activity of different pulp-capping materials was tested in this study; comparing the Ca(OH)2-based products with MTA-based ones. *Streptococcus mutans*, *Streptococcus salivarius* and *Streptococcus sanguis* microbial strains were selected. The aim of this study was to evaluate and compare, by the agar disc diffusion test, the antimicrobial activity of different pulp-capping materials: Dycal® (Dentsply Tulsa Dental), Calcicur® (Voco GmbH), Calcimol LC® (Voco GmbH), TheraCal LC® (Bisco Inc), MTA-Angelus® (Angelus), Biodentine® (Septodont).

## Material and Methods

Six pulp-capping materials were selected for this study: Dycal® (Dentsply Tulsa Dental), Calcicur® (Voco GmbH), Calcimol LC® (Voco GmbH), TheraCal LC® (Bisco Inc), MTA-Angelus® (Angelus), Biodentine® (Septodont).  Table 1 shows chemical composition of the materials tested: they were prepared in strict compliance to manufacturers’ instructions.

**Table 1:**
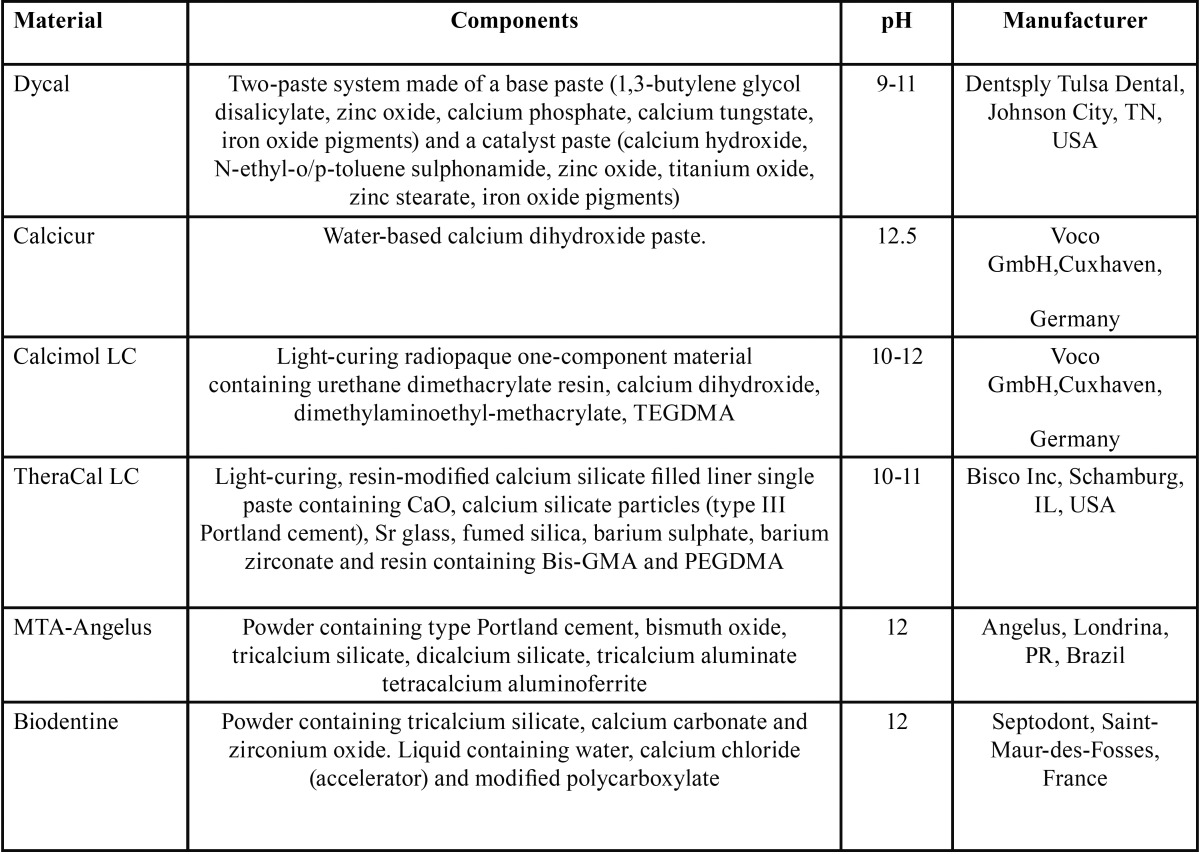
Characteristics of tested materials.

-Bacterial strains and growth conditions 

The *streptococcal strains* used in this study were from the Culture Collection of University of Goteborg (CCUG): *Streptococcus mutans* (CCUG 35176), *Streptococcus salivarius* (CCUG 11878), and *Streptococcus sanguis* (CCUG 17826). The cultures were grown and maintained in a Brain Heart Infusion (BHI, Difco, Detroit, MI, USA).* S. mutans* culture medium was supplemented with 10% (v/v) heat-inactivated horse blood serum (Oxoid, Rodano, Milano, Italy) to improve its growth. The culture of all bacterial strains was statically incubated for 16 h at 37 °C under aerobic conditions. This overnight culture, used as source for the experiments, was reduced at a final density of 1*1010 cells/ml as determined by comparing the OD600 of the sample with a standard curve relating OD600 to cell number.

-Agar disc diffusion test

Sterile paper discs (diameter: 6 mm, thickness: 1 mm) (Watman International, Maidstone, UK) were impregnated with 10 μl of each pulp capping material. All materials were prepared according to manufacturers’ recommendations. Then, BHI-agar plates were incubated with 1 x 107 cells/ml of an overnight culture of each streptococcal strain at 37°C for 20 minutes. The excess of bacterial suspension was removed from the plates and incubated with the paper disks impregnated with the pulp capping materials at 37°C for 24 h. The diameter of the halo formed around the paper disc (inhibition zone) was measured by the same operator in two perpendicular locations with a millimeter ruler (sliding callipers) with accuracy of 0.5 mm, after 24 h and 48 h. The size of the inhibition zone was calculated as follows: size of inhibition zone = (diameter of halo – diameter of specimen) x ½.

All the assays were conducted in triplicate and the results were recorded in terms of the average diameter of inhibition zone.

-Statistical analysis

The diameter of the growth inhibition zones was analyzed by Analysis of Variance (ANOVA). Firstly, data were assessed to be normal by means of Kolmogorov and Smirnov test. The Analysis of Variance was carried out and Tukey test was performed as post hoc. Significance was predetermined for *P*<0.001. Descriptive statistics, including mean, standard deviation, minimum, median and maximum, were calculated for each group tested. The analyses were conducted with Stata/SE 12.0 software.

## Results

**Figure 1:**
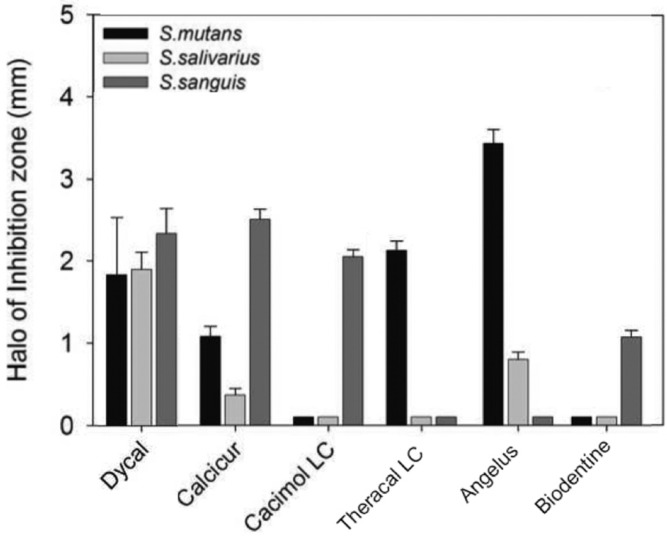
Antibacterial activity of the different pulp capping materials evaluated by agar diffusion test. Each paper disks impregnated with the different pulp capping materials were placed on agar plates previously incubated with the indicated streptococcal strains and incubate at 37°C for 24h. All the assays were conducted in triplicate and the results were recorded in terms of the average diameter of inhibition zone (mm). Error bars indicate standard errors of the means.

The antimicrobial activity of the tested pulp capping materials was evaluated with the agar disk diffusion test. As shown in figure 1, the results were quite different among the three streptococcal strains and the pulp capping materials. MTA-Angelus® (Angelus), TheraCal LC® (Bisco Inc), Dycal® (Dentsply Tulsa Dental) and Calcicur® (Voco GmbH) showed a decreasing antibacterial effect on *S.mutans*; only Dycal was effective against *S.salivarius*; Calcicur® (Voco GmbH), Dycal® (Dentsply Tulsa Dental), Calcimol LC® (Voco GmbH), followed by Biodentine® (Septodont) were effective on *S.sanguis*. Dycal® (Dentsply Tulsa Dental) was the only pulp capping material showing a fair antibacterial effect against all the three *streptococcal strains*. Descriptive statistic analysis for antibacterial properties are reported in  Table 2. For the investigation of the antibacterial properties the ANOVA showed the presence of significant differences among the various groups. Tukey test showed that when testing antibacterial activity with *Streptococcus salivarius* the highest growth inhibition values (*P*<0.001) were reported with Dycal® (Dentsply Tulsa Dental). MTA-Angelus® (Angelus) showed significantly lower values than Dycal and significantly higher values than all other pulp capping materials (*P*<0.001). Calcimol LC® (Voco GmbH) and TheraCal LC® (Bisco Inc) showed no significant differences among them (*P*>0.05) and all showed significantly lower values than Biodentine® (Septodont) and Calcicur® (Voco GmbH). When testing antibacterial activity with *Streptococcus sanguis* the highest growth inhibition values (*P*<0.001) were reported with Dycal® (Dentsply Tulsa Dental) and Calcicur® (Voco GmbH). The lowest growth inhibition values (*P*<0.001) were reported with MTA-Angelus® (Angelus) and TheraCal LC® (Bisco Inc). Biodentine® (Septodont) and Calcimol LC® (Voco GmbH) showed significantly lower values than Dycal® (Dentsply Tulsa Dental) and Calcicur® (Voco GmbH) and significantly higher values than all other materials tested (*P*<0.05). When testing antibacterial activity with *Streptococcus mutans* the highest growth inhibition values were reported with MTA-Angelus® (Angelus) (*P*<0.001). Significantly lower values were reported with TheraCal LC® (Bisco Inc) and Dycal® (Dentsply Tulsa Dental) that showed significantly higher values than Biodentine® (Septodont), Calcimol LC® (Voco GmbH) and Calcicur® (Voco GmbH) (*P*<0.05).

**Table 2:**
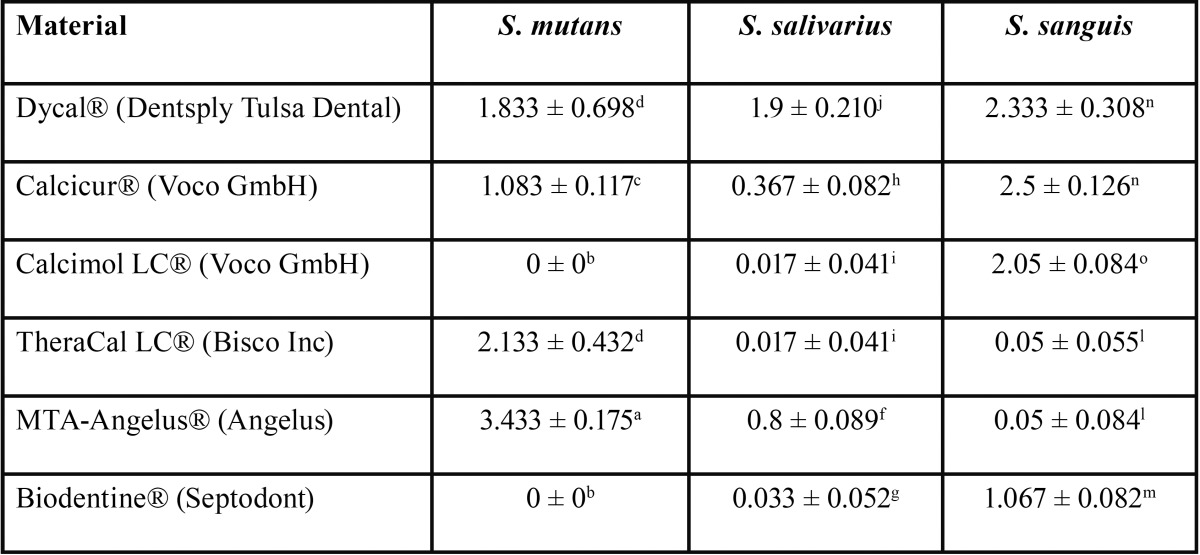
Mean and standard deviations of growth inhibition results (mm) of the different pulp capping materials.

## Discussion

In endodontic disease the primary aetiological agents is represented by microorganisms (13). The antibacterial activity of dental materials has been widely evaluated with the agar diffusion test (13-15). Agar diffusion test allows direct comparisons of materials against tested microorganisms. Nevertheless a great disadvantage of this method is represented by the impossibility of distinguishing between microbiostatic and microbicidal materials (16). The contact between the experimental material and agar, molecular weight, size and shape of the antimicrobial agent, load and concentration of the test material, agar gel viscosity and ionic concentration in relation to the medium must be considered, as relevant for the diffusion capacity of materials in agar.

Furthermore, the control and standardisation of the inoculation density, evaluation of results, selection of agar medium, incubation temperature of plates and reading point of inhibition haloes are restricting factors affecting the dynamics and variability of diffusion tests in an agar medium (17). Nevertheless, if most of these variables are carefully controlled, consistent and reproducible results may be obtained. In the present study Dycal® (Dentsply Tulsa Dental) and Calcicur® (Voco GmbH), both calcium hydroxide-based materials, showed antibacterial effects. Similar results were obtained for Calcimol LC® (Voco GmbH) and TheraCal LC® (Bisco Inc), both light-curing materials; even if the sensibility of the halo of inhibition zone was different among the species of microorganisms. MTA-based materials such as MTA-Angelus® (Angelus) and Biodentine® (Septodont) showed a variable effect against the different *Streptococci strains*.

These results confirmed the antibacterial activity of calcium hydroxide, as reported in previous studies (18). The antibacterial activity of Ca(OH)2 is based on the release of hydroxyl ions in solution (6). Hydroxyl ions are highly oxidant free radicals that show extreme reactivity with several biomolecules. The reactivity of hydroxyl ions is high and indiscriminate, diffusing from the generation site (7).

Differently, the antimicrobial effects of MTA-based materials is not well evaluated. MTA consists of 50-75 % (by weight) of calcium oxide and 15-25% of silicon dioxide. Blending these components, tricalcium silicate, dicalcium silicate, tricalcium aluminate and tetracalcium aluminoferrite were produced. The hydratation of the cement leads to the formation of a silicate hydrate gel. However it has been shown that, on hydration, MTA produces calcium hydroxide. Thus, it can be concluded that both MTA and calcium hydroxide may have a similar mechanism of action against bacterias (19).

Many studies have evaluated the effect of MTA on microorganisms, with conflicting results (9,20,21). Ribeiro *et al.* (22) focused that these variations might be due to the methodology used, such as aerobic and anaerobic incubations. On an aerobic atmosphere, MTA could generate reactive oxygen species which, as reported above, have antimicrobial activity similar to that obtained with calcium hydroxide. However, under anaerobic conditions, a decrease in the generation of radicals was observed (23).

According to Ribeiro *et al.* (22), in anaerobic conditions MTA is incapable of generating free radicals responsible for the antimicrobial effect on the different bacterial strains. Torabinejad *et al.* do not found MTA antibacterial effect against any of the strict anaerobic bacteria. However, as showed by our results, it is possible that MTA’s highly alkaline pH of 12.5 affords antimicrobial activity even in anaerobically condition (24).
